# General Dentists and Dental Specialists’ Knowledge of Treatment, Diagnosis, Referral, and Risk Factors of Obstructive Sleep Apnea: A Systematic Review

**DOI:** 10.3390/dj13050187

**Published:** 2025-04-24

**Authors:** Shahad A. Alkharouby, Sumayyah L. Alkhudhayri, Shahad L. Alhassani, Hamed S. Alghamdi, Rashed A. Alsahafi, Nivetha Mariappan, Mohammed A. Barashi, Hesham A. Alhazmi

**Affiliations:** 1 College of Dentistry, Umm Al-Qura University, Makkah 24211, Saudi Arabia; sumaya.l1999@gmail.com (S.L.A.); shahadalhassani9@gmail.com (S.L.A.); 2 Department of Oral and Maxillofacial Surgery and Diagnostic Science, College of Dentistry, Umm Al-Qura University, Makkah 24211, Saudi Arabia; hsghamdi@uqu.edu.sa; 3 Department of Restorative Dental Sciences, College of Dentistry, Umm Al-Qura University, Makkah 24211, Saudi Arabia; rasahafi@uqu.edu.sa; 4 Harvard School of Dental Medicine, Boston, MA 02115, USA; nivetha_mariappan@hsdm.harvard.edu; 5 Department of Preventive Dentistry, College of Dentistry, Umm Al-Qura University, Makkah 24211, Saudi Arabia; mabarashi@uqu.edu.sa (M.A.B.); haahazmi@uqu.edu.sa (H.A.A.)

**Keywords:** obstructive sleep apnea (OSA), knowledge, dental specialists, general dentists

## Abstract

**Objectives:** This systematic review aimed to evaluate general dentists and dental specialists’ knowledge regarding obstructive sleep apnea (OSA) diagnosis, referral, risk factors, and treatment. **Methods:** A systematic search of databases, including Web of Science, PubMed, and ProQuest, was conducted for studies published up to 25 September 2023, following the Preferred Reporting Items for Systematic Reviews and Meta-Analyses guidelines. Inclusion criteria included cross-sectional studies that assessed the knowledge of general dentists or dental specialists. A quality assessment was performed using the Newcastle–Ottawa Quality Assessment Scale. **Results:** The seven included studies demonstrated varied knowledge levels among respondents regarding polysomnography as the gold standard for diagnosing OSA, with percentages ranging from 40.18% to 90%. While recognition of craniofacial structure as a risk factor for OSA was consistently high, knowledge about body weight as a risk factor varied. Additionally, the understanding of continuous positive airway pressure as the standard treatment showed discrepancies across the studies. **Conclusions:** Given that some of the included articles displayed a moderate to high risk of bias, the results highlight the varying levels of OSA knowledge among dentists and specialists across the studies. This indicates a potential need for targeted educational programs to improve their understanding and management of OSA.

## 1. Introduction

One of the most common sleep-related breathing disorders that disrupts regular sleep cycles is obstructive sleep apnea (OSA) [1], a clinical disorder that induces recurrent episodes of complete or partial obstruction of the upper airway for more than 10 s despite persistent respiratory efforts [2]. Dentists often serve as the first point of contact for undiagnosed cases of OSA and play a role in its early diagnosis [3]. Dentists can use polysomnography (PSG) for diagnosis, which is considered the gold standard for evaluating sleep disorders [4]. PSG is a monitored, eight-hour sleep study performed in a laboratory with standardized scoring criteria for OSA-related respiratory episodes and involves a comprehensive procedure that records brain activity (electroencephalogram), eye movements (electro-oculogram), muscle activity (electromyogram), heart activity (electrocardiogram), and pulse oximetry, as well as airflow and respiratory effort to identify the underlying causes of sleep disturbances [4].

Several risk factors have been reported for OSA, including anomalies in craniofacial or oropharyngeal anatomy, as well as obesity [5]. Regarding the former, while sleeping, the tension in the dilator muscles that help keep the airway open can decrease. For the latter, the relative constriction of the airway lumen associated with obesity increases the chances of obstruction [6]. Furthermore, every rise in body mass index (BMI) beyond the 50th percentile correlates with approximately a 10% higher risk for OSA [7]. Another risk factor for OSA is maxillofacial abnormalities. The presence of various craniofacial anatomical factors reduces the patency of the upper airway and may contribute to the development of OSA [2]. Other risk factors include age, with individuals over the age of 40 having a higher prevalence of OSA. Gender is also a reported risk factor, with males experiencing a greater prevalence of OSA than females. Additionally, lifestyle choices may play a role in increasing the likelihood of developing OSA, with alcohol consumption and smoking being associated with the condition [5].

The American Academy of Dental Sleep Medicine (AADSM) advises dentists to assess patients for sleep-related breathing disorders using questionnaires and airway evaluations. It is essential for dentists to provide timely and consistent follow-up care while working closely with physicians. Furthermore, the AADSM stated that it is the responsibility of physicians to prescribe the most appropriate treatment option [8]. The recommended treatment for patients with moderate to severe OSA is continuous positive airway pressure (CPAP). This therapy helps decrease the Apnea-Hypopnea Index and modifies sleep patterns, leading to an improvement in OSA symptoms [9].

A proper understanding of OSA is essential for dental providers, as untreated OSA has a range of repercussions that can impair the health of patients. Some of these consequences involve negative impacts on the cardiovascular and metabolic systems, such as hypertension, increased risk for insulin-resistant diabetes, and acute myocardial infarction [2]. A lack of knowledge regarding OSA symptoms, risk factors, and available treatment options can lead to underdiagnosis and delayed treatment. The goal of this systematic review was to assess the knowledge of general dentists and dental specialists regarding the diagnosis, treatment, risk factors, and referral of patients with OSA.

## 2. Materials and Methods

### 2.1. Protocol and Search Strategy

This systematic review was carried out in accordance with the Preferred Reporting Items for Systematic Reviews and Meta-Analyses (PRISMA) guidelines [10]. Focused PICO (Population, Intervention, Comparison, and Outcome) questions were used to assess practitioner dentists and dental specialists’ knowledge of the risk factors, diagnosis, referral, and treatment of OSA. A systematic search of databases, including Web of Science, PubMed, and ProQuest, was conducted for studies published up to 25 September 2023, using related terms such as “knowledge”, “aware*”, “attitude*”, “dent*”, and “obstructive sleep apnea”. A manual search was carried out for related publications. Our search was limited to studies published in English. Two of the authors assessed the title/abstracts independently to determine whether they met the inclusion criteria. In cases of disagreement, a third author was involved in all these processes. The full text of the selected articles was checked for further information.

### 2.2. Selection of Studies, Data Extraction, and Eligibility Criteria

From the selected studies, two of the authors extracted data such as the name of the authors, the year of publication, the total sample size, and the number of participating dentists and specialists. In cases of disagreement, a third author was involved in all these processes.

Eligibility criteria included the following:

1. Cross-sectional studies;
2. Studies including an assessment of general dentists or dental specialists’ knowledge;
3. Studies including questions that assess the knowledge (treatment, diagnosis, referral, and risk factors) of OSA;
4. Studies written in English.

### 2.3. Quality Assessment Tools

The scale used for the quality assessments in this study was adapted from the Newcastle–Ottawa Quality Assessment Scale for cross-sectional studies [11]. The scale consisted of nine items, each with a maximum of one point, except for two items that had a maximum of two points. The assessment scale was performed by two authors (SU and SH) separately, and a third author was involved only in the case of any disagreements.

## 3. Results

### 3.1. Study Characteristics

**Figure 1:**
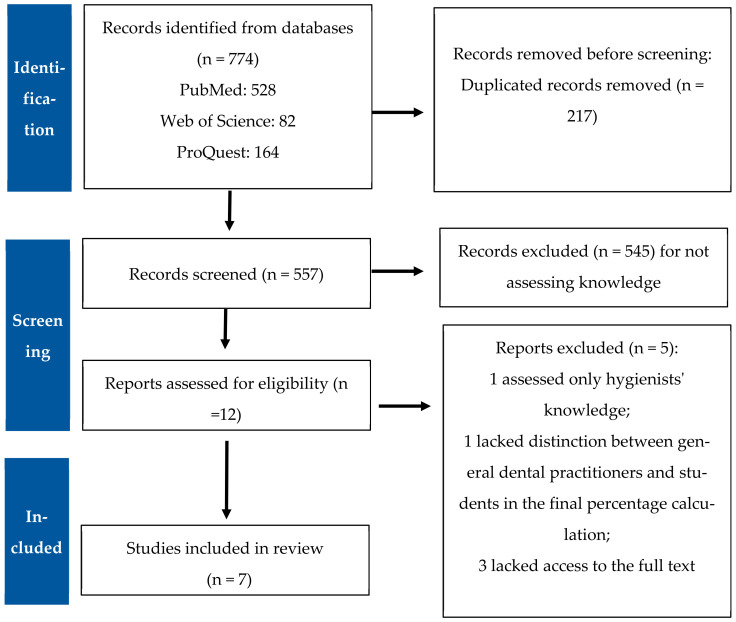
PRISMA flowchart for included studies and exclusion reasons.

The three databases were searched, and 774 studies were identified, among which 217 duplicates were found. After checking the remaining 557 titles and abstracts, 545 studies were found to not match the inclusion criteria. Thereafter, of the 12 remaining publications that were chosen for full-text evaluation, 5 were excluded because they either lacked access to the full text or an assessment of dental students or dental hygienists’ knowledge [12,13,14,15] (Figure 1). Thus, a total of seven cross-sectional studies were included in this review. The included studies were conducted in different countries (Saudi Arabia: 2; India: 2; Lithuania: 1; Finland: 1; and United States: 1). Three studies did not stratify their results according to general dentists and dental specialists [16,17,18], while four studies differentiated between general dentists and dental specialists [2,3,19,20].

### 3.2. Assessment of Knowledge Regarding Diagnosis, Treatment, Risk Factors, and Referral for OSA Patients

#### 3.2.1. Knowledge of Using PSG as a Gold Standard in Diagnosing OSA

Five studies assessed the use of PSG as the gold standard for diagnosing OSA. The reported knowledge levels varied across studies. Sawan et al. reported that 48.40% of respondents in Saudi Arabia chose PSG as the gold standard to diagnose OSA [3], and Alzahrani et al. reported a similar percentage, with 44.8% among the dentists [2], while Kale et al. showed a slightly lower percentage, with 40.18% of participants acknowledging PSG as the gold standard [16]. In contrast, Bian reported a higher percentage, with 90% of participants recognizing overnight PSG as the gold standard for diagnosing moderate or severe OSA (Table 1) [18].

#### 3.2.2. Knowledge of Risk Factors for OSA

While most studies reported a higher knowledge prevalence of craniofacial structure as a risk factor, knowledge of body weight as a risk factor was more inconsistent. For instance, Sawan et al. found that 81.11% of the respondents acknowledged weight as a risk factor for OSA [3], whereas Kale et al. reported a lower knowledge prevalence of 33.92% [16]. Similarly, while Alzahrani et al. found that 73.4% of dentists disagreed that OSA is more prevalent in individuals with a low body mass index [2], Jokubauskas et al. and Vuorjoki-Ranta et al. reported that 68% and 47% of participants, respectively, reported that OSA occurs less frequently in individuals with normal weight [19,20]. In contrast, the knowledge of craniofacial structure as a risk factor was consistently higher across studies. Nair et al. reported that 97.4% of specialists reported abnormal maxilla and mandibular development as a risk factor [17]. Similarly, 77.68% of respondents in Kale et al.’s study and 70% in Jokubauskas et al.’s study recognized its association with OSA [16,19]. The highest knowledge level was reported by Vuorjoki-Ranta et al., where 98.5% of participants acknowledged craniofacial structure’s role in OSA (Table 2) [20].

#### 3.2.3. Knowledge of Using CPAP as the Gold Standard Treatment for OSA

Six studies assessed the knowledge of CPAP as the gold standard treatment for OSA, reporting varying knowledge percentages. Vuorjoki-Ranta et al. reported the highest knowledge percentage, at 99.3% [20], followed by Nair et al., with 46.1% [17]. Sawan et al. and Jokubauskas et al. reported similar percentages of 36.44% and 36.5%, respectively [3,19]. Alzahrani et al. found a lower percentage of 28.6% among respondents (Table 3) [2].

#### 3.2.4. Knowledge of Referral of Patients with OSA

Six studies assessed referral percentages for OSA cases, showing some variation. Nair et al. reported the highest referral percentage, with 96.1% of respondents referring patients to specialists such as otolaryngologists and pulmonologists [17], followed by Kale et al., with a referral percentage of 88.39% among those who agreed that dentists should screen for OSA and refer patients with abnormal anatomical structures [16]. Alzahrani et al. also reported a high referral percentage of 84.4% among dentists, recognizing the importance of referring high-risk patients to sleep physicians [2]. Vuorjoki-Ranta et al. reported that 59% of the dentists consulted OSA specialists when they suspected the condition [20]. In a similar vein, Sawan et al. reported that 25.11% of dentists never consulted physicians regarding OSA [3], and Bian et al. reported the lowest referral percentage, with 54% of dentists never referring suspected OSA cases (Table 4) [18].

### 3.3. Quality Assessment

Table 5 shows the Newcastle–Ottawa scores for all seven included articles, ranging from 3 to 7. Out of nine points, Sawan et al. received four points [3], Nair et al. and Jokubauskas et al. received seven [17,19], Alzahrani et al. received six [2], Kale et al. and Vuorjoki-Ranta et al. received five [16,20], and Bian’s study received three [18].

## 4. Discussion

This systematic review assessed general dental practitioners and dental specialists’ knowledge of OSA diagnosis, risk factors, referral, and treatment. Globally, OSA affects approximately 936 million adults, with 425 million experiencing moderate to severe cases [21]. However, OSA is often undiagnosed because physicians may have difficulty recognizing its signs and symptoms [22]. While obesity is a major risk factor, others include male sex, age, craniofacial abnormalities, smoking, and alcohol consumption [23].

The reviewed studies highlighted varying levels of knowledge on the use of PSG to diagnose OSA. While some research indicated a strong recognition of PSG as the standard diagnostic tool, others showed less knowledge. For instance, Bian reported a high knowledge percentage of 90% [18], while Kale et al. found that only 40.18% of respondents recognized PSG as the standard diagnostic tool [16]. These discrepancies underscore the need for targeted education and training to enhance healthcare providers’ understanding, beginning in dental and medical schools. Alharbi et al. noted that 38% of medical and 25% of dental students in Saudi Arabia had good knowledge of OSA, emphasizing the importance of increasing dental practitioners’ role in OSA diagnosis and management [24].

Several studies assessed obesity as a factor in OSA, and the findings varied across studies. Sawan et al. [3] and Alzahrani et al. [2], both from Saudi Arabia, reported that 81.11% and 73.40% of respondents, respectively, acknowledged weight as a risk factor. Furthermore, Jokubauskas et al., from Lithuania, found that 68% of dentists agreed that OSA is less common in individuals with normal body weight [19]. However, Kale et al., from India, indicated that only 33.92% considered obesity to be a contributing factor [16], while Vuorjoki-Ranta et al., from Finland, suggested that 47% of respondents agreed that people with normal weight have OSA less often [20]. The differences in percentages among these studies may stem from cultural perceptions, sample demographics, and each study’s specific focus, indicating the complexity of the relationship between obesity and OSA. The consistency observed in the Saudi Arabian and Lithuanian studies with respect to body weight and craniofacial structures’ influence on OSA underscores these factors’ importance in diagnosing and managing OSA. Practitioners in these regions might be better informed about the primary risk factors. In a study conducted in the United States, Californian dentists scored around 63.4% in OSA knowledge, while physicians scored higher, particularly in diagnosis and treatment, highlighting a knowledge gap [25]. The discrepancies observed in the Indian and Finnish studies suggest a need for more uniform educational initiatives and guidelines across regions to enhance understanding of OSA risk factors.

Several studies included in this review indicated gaps in interdisciplinary collaboration between dentists and other healthcare specialists. For instance, Sawan et al. reported that 25% of dentists in Saudi Arabia never consulted physicians about suspected OSA cases, which highlights a potential barrier to timely diagnosis and management [3]. In contrast, Nair et al. found that the majority (96.1%) of dentists who participated in their study referred suspected OSA patients to otolaryngologists, pulmonologists, sleep medicine physicians, or dental sleep medicine specialists [17]. Moreover, regional differences were evident in referral practices. Alzahrani et al. demonstrated that 84.4% of dentists in Saudi Arabia referred high-risk potential OSA patients to sleep physicians, indicating a relatively high knowledge and proactive approach compared with other regions [2]. Furthermore, studies such as that of Kale et al. in India highlighted variability in dentists’ recognition of anatomical risk factors’ association with OSA, as well as their willingness to further investigate such cases or to refer them for further evaluation [16].

In Saudi Arabia, Sawan et al. found that 36.44% of dentists considered CPAP to be the gold standard treatment for sleep apnea [3], and Alzahrani et al. reported that 28.6% of Saudi Arabian dentists felt confident in prescribing oral appliances for mild to moderate OSA [2]. In India, Nair et al. reported that 46.2% of dentists identified the appropriate treatment options for OSA correctly [16,17]. In a study that was conducted in India to assess the management of OSA, researchers found that 20% of students and dental healthcare providers actively prescribed oral appliances [26]. Furthermore, Lin et al. reported that over 50% of orthodontic professionals felt that they lacked knowledge of OSA management [14]. In a study that included 324 final-year medical students across three institutes in India, only 16.66% were confident that they could administer CPAP therapy [27]. Similarly, a study conducted in Malaysia found that 15.5% of dental students agreed that they felt confident in their ability to manage OSA patients [28]. Another study that compared graduating Saudi medical and dental students’ knowledge levels and attitudes found that around 38.0% of medical participants had good knowledge of pediatric OSA compared with the 25.2% from the dental field [24]. In 2023, a study surveyed patients at the Unit of Orthodontics and Sleep Dentistry and found that only 66.3% of the patients reported familiarity with CPAP therapy, despite it being the first-line treatment for OSA [29]. In addition, a study conducted in Saudi Arabia that assessed patients’ families’ knowledge and awareness of OSA noted that most participants were unaware of sleep disorders unless they had family members with diagnosed cases, indicating a need for public awareness campaigns [30]. Overall, the reviewed studies revealed both similarities and differences in interdisciplinary collaboration and treatment approaches to OSA among dentists across different regions. A commonality is the widespread recognition of CPAP as a key treatment, which reflects a broad consensus on its importance [31]. However, regional differences emerged in referral practices and attitudes toward treatment modalities [32]. While Indian and Saudi Arabian dentists often refer suspected OSA cases to specialists, how consistently these referrals are made varies. These variations are likely influenced by differences in healthcare systems, professional training, and regional awareness of OSA management practices [2,3,16,17]. Overall, these findings highlight the need for improved education and standardized practices to enhance interdisciplinary collaboration and treatment efficacy for OSA globally [33].

This study is the first systematic review to assess dentists’ knowledge of OSA by evaluating four key components: the gold standards of treatment and diagnosis, the risk factors, and the referral process. However, there are notable limitations. First, all studies included were cross-sectional. In addition, the quality assessment indicated that some articles displayed a moderate to high risk of bias. Furthermore, most studies did not differentiate between general dentists and specialists’ knowledge levels, which may affect the interpretation of the results overall. Future research that assesses dentists’ knowledge of OSA should consider differentiating between general dentists and specialists. This differentiation could facilitate the development of tailored educational programs designed to advance their understanding of OSA. In addition, employing more robust study designs, such as longitudinal studies, could increase the results’ reliability and provide deeper insights into educational interventions’ effectiveness. By addressing these aspects, future studies can contribute to a more comprehensive understanding of knowledge gaps and improve the management of OSA in dental practice overall.

## 5. Conclusions

This systematic review showed varying levels of knowledge about OSA among general dentists and specialists across the included studies. Some studies indicated that less than half of the dentists recognized PSG as the gold standard diagnostic tool. Most included studies reported that craniofacial structure plays a role in OSA. However, the knowledge regarding treatment options and the necessity for referrals for OSA varied among the studies, suggesting potential gaps in understanding. These findings highlight the need for focused educational programs to enhance the knowledge and management of OSA among dental professionals overall.

## Figures and Tables

**Table 1:** Characteristics of the studies and their major diagnostic findings using polysomnography as the gold standard method for treating cases with OSA.

Author	Year	Country	Sample Size	Major Findings
Sawan et al. [3]	2023	Saudi Arabia	450	218 (48.40%) general dentists and specialists chose PSG * as the gold standard for diagnosis.
Nair et al. [17]	2023	India	121	77 (63.6%) specialists chose PSG * as the gold standard for diagnosis.
Alzahrani et al. [2,16]	2022	Saudi Arabia	191	85 (44.5%) general dentists and dental consultants did not know how to diagnose OSA **.
Kale et al. [16,18]	2020	India	112	45 (40.18%) dentists correctly chose polysomnography as the gold standard for OSA diagnosis.
Bian [18]	2006	United States	192	173 (90%) acknowledged the significance of overnight polysomnography as the standard method in sleep studies for diagnosing moderate or severe OSA **.

PSG *: polysomnography. OSA **: obstructive sleep apnea.

**Table 2:** Characteristics of the studies and their major findings for risk factors of OSA (weight and craniofacial structure).

Author	Year	Country	Sample Size	Major Findings
Sawan et al. [3,17]	2023	Saudi Arabia	450	365 (81.11%) agreed that weight is considered a risk factor for OSA *.
Nair et al. [17]	2023	India	121	118 (97.5%) agreed that abnormal maxilla and mandibular development is associated with OSA *.
Alzahrani et al. [2]	2022	Saudi Arabia	191	140 (73.30%) disagreed that OSA * is more common in people with a low body mass index. 147 (77.00%) agreed that maxillofacial abnormalities increase the risk of OSA *.
Kale et al. [16]	2020	India	112	38 (33.92%) agreed that obesity contributes to OSA *. 87 (77.68%) agreed that abnormal maxilla and mandibular development can be a risk factor for OSA *.
Jokubauskas et al. [19]	2019	Lithuania	353	240 (68%) agreed that OSA * is less common in people with normal body weight. 247 (70%) agreed that craniofacial structure is relevant regarding OSA *.
Vuorjoki-Ranta et al. [20]	2016	Finland	134	63 (47%) agreed that people with normal weight have OSA * less often. 132 (98.5%) agreed that craniofacial structure plays a role in OSA *.

OSA *: obstructive sleep apnea.

**Table 3:** Characteristics of the studies and their major treatment findings when using CPAP for OSA.

Author	Year	Country	Sample Size	Major Findings
Sawan et al. [3]	2023	Saudi Arabia	450	164 (36.44%) acknowledge CPAP* as the gold standard treatment for sleep apnea.
Nair et al. [17]	2023	India	121	56 (46.2%) selected the correct choice of treatment of OSA **.
Alzahrani et al. [2]	2022	Saudi Arabia	191	54 (28.6%) believe that dentists can prescribe oral appliances to treat mild and moderate OSA **.
Jokubauskas et al. [19]	2019	Lithuania	353	129 (36.5%) acknowledge CPAP* as a notable option for treating OSA *.
Vuorjoki-Ranta et al. [20]	2016	Finland	134	133 (99.3%) acknowledge CPAP as an effective method for treating OSA.

CPAP*: continuous positive airway pressure. OSA **: obstructive sleep apnea.

**Table 4:** Characteristics of the studies and their major referral findings for OSA cases.

Author	Year	Country	Sample Size	Major Findings
Sawan et al. [3]	2023	Saudi Arabia	450	113 (25.11%) responding dentists never consulted with physicians.
Nair et al. [17]	2023	India	121	116 (96.1%) respondents referred patients to otolaryngologists, pulmonologists, sleep medicine physicians, or dental sleep medicine specialists for suspicion of OSA *.
Alzahrani et al. [2]	2022	Saudi Arabia	191	161 (84.4%) respondents acknowledge that a dentist plays a role in referring potential patients who are at high risk of OSA * to a sleep physician.
Kale et al. [16]	2020	India	112	99 (88.39%) respondents agreed or strongly agreed that a dentist who encounters abnormal anatomical oral structures should conduct further investigations for OSA * and refer the patient to a sleep physician.
Vuorjoki-Ranta et al. [20]	2016	Finland	134	79 (59%) responding dentists consult an OSA * specialist when they suspect OSA.
Bian [18]	2006	United States	192	104 (54%) responding dentists indicated they never consulted with physicians for suspected OSA * during their practice.

OSA *: obstructive sleep apnea.

**Table 5:** The Newcastle–Ottawa checklist and quality assessment ^*^.

Author	Item 1	Item 2	Item 3	Item 4	Item 5	Item 6	Item 7	Total Score
Sawan et al. [3]	0	0	*	*	0	*	*	4
Nair et al. [17]	*	*	*	*	*	*	*	7
Alzahrani et al. [2]	*	*	*	*	0	*	*	6
Kale et al. [16]	*	0	*	*	0	*	*	5
Jokubauskas et al. [19]	*	*	*	*	*	*	*	7
Vuorjoki-Ranta et al. [20]	*	*	*	*	*	*	*	5
Bian [18]	0	0	*	0	0	*	*	3

* Item 1 is the representativeness of the cases (maximum score of one), item 2 refers to the sample size (maximum score of one), item 3 addresses the non-response rate (maximum score of one), item 4 pertains to the screening/surveillance tool ascertainment (maximum score of two), item 5 evaluates comparability (maximum score of one), item 6 involves the outcome assessment (maximum score of two), and item 7 considers the used statistical test (maximum score of one).

## Abbreviations

OSA	Obstructive sleep apnea
PSG	Polysomnography
CPAP	Continuous positive airway pressure
